# Integration of genome-wide association studies and gene coexpression networks unveils promising soybean resistance genes against five common fungal pathogens

**DOI:** 10.1038/s41598-021-03864-x

**Published:** 2021-12-27

**Authors:** Fabricio Almeida-Silva, Thiago M. Venancio

**Affiliations:** Laboratório de Química e Função de Proteínas e Peptídeos, Centro de Biociências e Biotecnologia, Universidade Estadual do Norte Fluminense Darcy Ribeiro, Av. Alberto Lamego 2000, P5, sala 217, Campos dos Goytacazes, RJ Brazil

**Keywords:** Computational biology and bioinformatics, Plant biotechnology

## Abstract

Soybean is one of the most important legume crops worldwide. However, soybean yield is dramatically affected by fungal diseases, leading to economic losses of billions of dollars yearly. Here, we integrated publicly available genome-wide association studies and transcriptomic data to prioritize candidate genes associated with resistance to *Cadophora gregata*, *Fusarium graminearum*, *Fusarium virguliforme, Macrophomina phaseolina*, and *Phakopsora pachyrhizi*. We identified 188, 56, 11, 8, and 3 high-confidence candidates for resistance to *F. virguliforme, F. graminearum, C. gregata, M. phaseolina and P. pachyrhizi*, respectively. The prioritized candidate genes are highly conserved in the pangenome of cultivated soybeans and are heavily biased towards fungal species-specific defense responses. The vast majority of the prioritized candidate resistance genes are related to plant immunity processes, such as recognition, signaling, oxidative stress, systemic acquired resistance, and physical defense. Based on the number of resistance alleles, we selected the five most resistant accessions against each fungal species in the soybean USDA germplasm. Interestingly, the most resistant accessions do not reach the maximum theoretical resistance potential. Hence, they can be further improved to increase resistance in breeding programs or through genetic engineering. Finally, the coexpression network generated here is available in a user-friendly web application (https://soyfungigcn.venanciogroup.uenf.br/) and an R/Shiny package (https://github.com/almeidasilvaf/SoyFungiGCN) that serve as a public resource to explore soybean-pathogenic fungi interactions at the transcriptional level.

## Introduction

Soybean (*Glycine max* (L.) Merr.) is a major legume crop worldwide, contributing to global food security and economy. However, soybean yield is significantly affected by diseases, with an estimated economic loss of 95.8 billion dollars from 1996 to 2006 in the US^1^. Most of the yield loss has been linked to foliar and stem/root diseases, which are mostly caused by phytopathogenic fungi^1^. Fungal diseases, such as sudden death syndrome, Fusarium wilt, brown stem rot and asian rust, can impact soybean crops through leaf damage, necrosis, chlorosis, and death^1–3^.

Over the past decade, several genome-wide association studies (GWAS) have uncovered multiple single-nucleotide polymorphisms (SNPs) associated with resistance to pathogenic fungi in soybean populations^3–9^. Nevertheless, GWAS often fail to accurately pinpoint the causative genes^10^. GWAS limitations are particularly challenging for self-pollinating plants (e.g., soybean) because of limited recombination and strong linkage disequilibrium between causative and non-causative variants^11^. Such limitations ultimately lead to large genetic intervals with several genes, hindering causative gene identification. Because of the exponential accumulation of genomic and transcriptomic data in public databases^12–16^, integrative analyses to prioritize candidate genes have become a promising approach. This strategy consists in investigating the transcriptional patterns of all the genes near a significant SNP. Hence, the combination of multiple sources of evidence can result in richer and narrower sets of high-confidence candidate genes for downstream experimental validation towards biotechnological applications.

Here, we integrated multiple publicly available RNA-seq and GWAS datasets to identify high-confidence candidate genes for resistance to five phytopathogenic fungi. The prioritized resistance genes are species-specific and highly conserved in the pangenome of cultivated soybeans. The candidate resistance genes against each species are involved in various immunity-related processes, such as recognition, signaling, oxidative stress, and apoptosis. Finally, we highlighted the five most resistant accessions against each fungal species in the USDA germplasm, uncovering important information for breeding programs and genetic engineering initiatives. Finally, the coexpression network resulting from this work was also made available as a publicly available web application (https://soyfungigcn.venanciogroup.uenf.br/) and R/Shiny package (https://github.com/almeidasilvaf/SoyFungiGCN).

## Materials and methods

### Curation of resistance-associated SNPs and pan-genome data

SNPs that contribute to resistance against phytopathogenic fungi were manually curated from the scientific literature (Table 1; Supplementary Table S1). SNPs that were identified using the Gmax_a1.v1 genome were converted to their corresponding sites in the Gmax_a2.v1 assembly using the .vcf files for both assemblies available at Soybase^17^. A matrix of gene presence/absence variation (PAV) in the pan-genome of cultivated soybeans (*n* = 204 genomes from 24 countries and 5 continents) was obtained from the Supplementary Data in^18^.

**Table 1:** GWAS included in this work.

References	Pathogen	Resistance SNPs
^8^	*F. graminearum*	12
^32^	*F. virguliforme*	8
^9^	*C. gregata/F. virguliforme/P. pachyrhizi*	2/1/2
^7^	*F. virguliforme*	32
^33^	*F. virguliforme*	27
^34^	*M. phaseolina*	4
^35^	*M. phaseolina*	12
^3^	*C. gregata*	7

### Transcriptome data

Gene expression estimates in transcripts per million mapped reads (TPM, Kallisto estimation) were retrieved from the Soybean Expression Atlas^19^. Additional RNA-seq samples comprising soybean tissues infected with fungal pathogens were retrieved from a recent publication from our group^20^. We filtered the SNP and transcriptome datasets to keep only fungal species that were represented by both data sources. A total of 150 RNA-seq samples from soybean tissues infected with fungal pathogens were selected (Supplementary Table S2). Finally, genes with median expression values lower than 5 were excluded to attenuate noise, resulting in an 18,748 × 150 gene expression matrix for downstream analyses.

### Selection of guide genes

MapMan annotations for soybean genes were retrieved from the PLAZA 3.0 Dicots database^21^. Genes assigned to defense-related pathways (e.g., pathogenesis-related proteins, lignin biosynthesis, oxidative stress, and phytohormone regulation) were used as guides (Supplementary Table S3).

### Candidate gene mining and functional analyses

Gene expression data were adjusted for confounding artifacts and quantile normalized with the R package BioNERO^22^. An unsigned coexpression network was inferred with BioNERO using Pearson’s r as correlation. All genes located in a 2 Mb sliding window relative to each SNP were selected as putative candidates, as previously proposed^23^. Candidate genes were prioritized using the algorithm implemented in the R package cageminer^24^, with an r_pb_ threshold of 0.2 for gene significance (gene-trait correlation). Enrichment analyses were also performed with BioNERO, using functional annotations from the PLAZA 4.0 database^25^. To rank the prioritized candidates, they were given scores using the formula:

$$S = r_{pb} \kappa$$

where

- $r_{pb}$ = point-biserial correlation coefficient (cageminer algorithm)
- $\kappa = 2$ if the gene is a transcription factor
- $\kappa = 2$ if the gene is a hub
- $\kappa = 3$ if the gene is a hub and a transcription factor
- $\kappa = 1$ if the gene is neither a hub nor a transcription factor.

### Selection of most resistant accessions from the USDA germplasm

The VCF file with genotypic information for all accessions in the USDA germplasm was downloaded from Soybase^17^. For each locus, scores 0, 1, or 2 were attributed if accessions had 0, 1, or 2 beneficial SNPs (effect size > 0), respectively, whereas scores 2, 1, or 0 were attributed if accessions had 0, 1, or 2 deleterious SNPs (effect size < 0). Total resistance scores for each accession were calculated as the sum of scores S_i_ for all *n* loci as follows:

$$S_{total} = \mathop \sum \limits_{i = 1}^{n} S_{i} ,\;where\;S_{i} = \left\{ {0,1,2} \right\}$$

Total resistance scores were ranked from highest to lowest, and ranks were used to select the most resistant accessions. The resistance potential of the best accessions was calculated as a ratio of the attributed scores to the theoretical maximum score (all beneficial SNPs and no deleterious SNPs).

## Results and discussion

### Data summary and genomic distribution of SNPs

**Figure 1:**
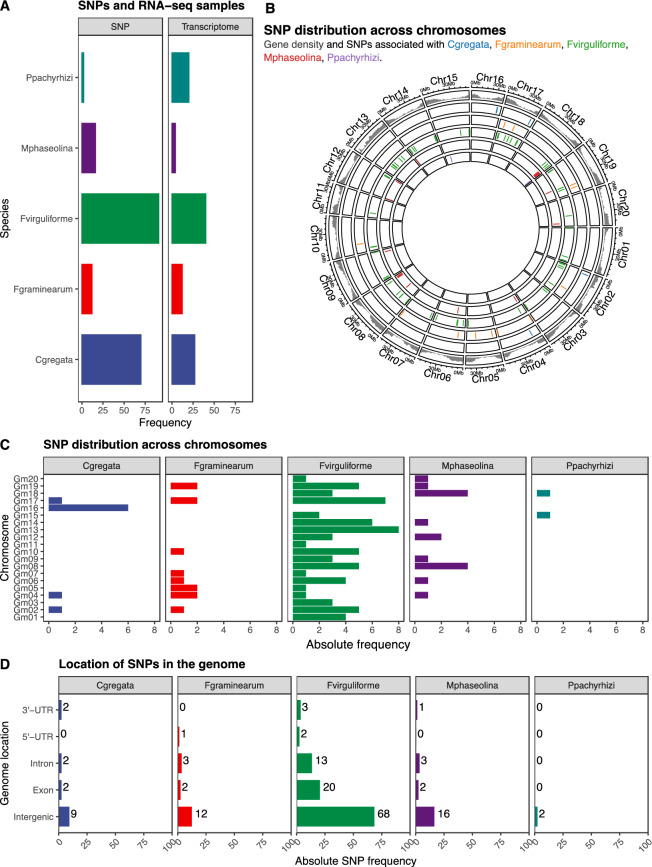
Data summary and genomic distribution of SNPs. (**A**) Frequency of SNPs and RNA-seq samples included in this study. (**B**) Genomic coordinates of resistance SNPs against each fungal pathogen. The outer track represents gene density, whereas inner tracks represent the SNP positions for each species. (**C**) SNP distribution across chromosomes. Overall, there is an uneven distribution of SNPs across chromosomes. (**D**) Genomic location of SNPs. Most SNPs are located in intergenic regions.

After filtering the datasets to keep only fungal species represented by both SNP and transcriptome information, we kept five common phytopathogenic fungi: *Cadophora gregata*, *Fusarium graminearum*, *Fusarium virguliforme*, *Macrophomina phaseolina*, and *Phakopsora pachyrhizi* (Fig. 1A). Overall, SNPs were located in gene-rich regions of the genome (Fig. 1B). SNPs were unevenly distributed across chromosomes, except for *F. virguliforme* (Fig. 1C). Further, we found that most SNPs were located in intergenic regions (Fig. 1D). Hence, predicting SNP effect on genes would not be suitable for this trait.

### Candidate gene mining reveals a highly species-specific immune response

**Figure 2:**
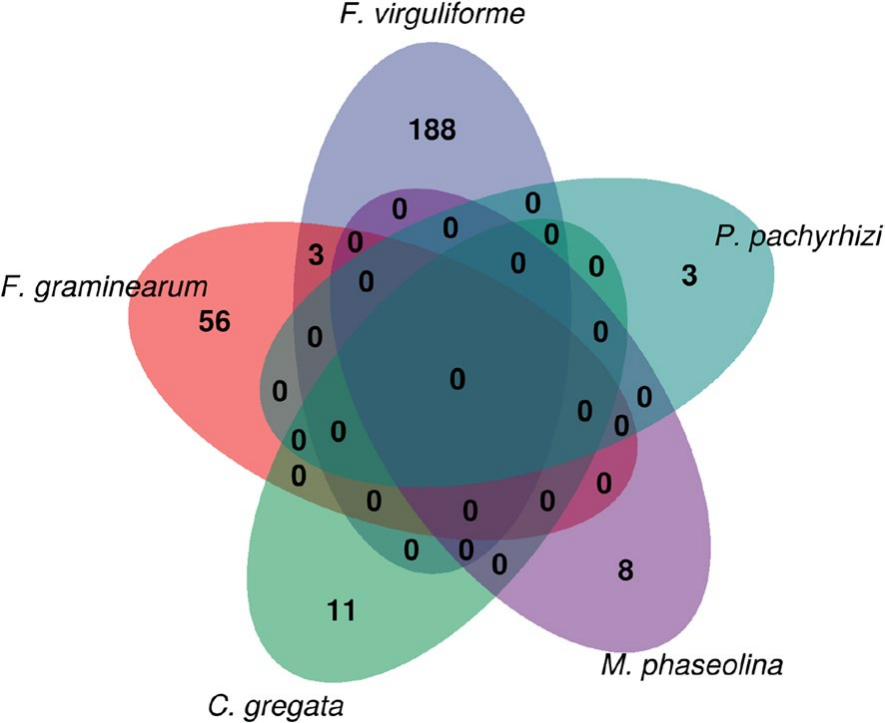
Venn diagram of prioritized candidate resistance genes against each species. The diagram demonstrates a high species-specific response to each pathogen, as genes are mostly not shared. Only three genes are shared between *F. graminearum* and *F. virguliforme*, suggesting some conservation at the genus level.

Using defense-related genes as guides, the cageminer algorithm identified 188, 56, 11, 8, and 3 high-confidence genes for *F. virguliforme, F. graminearum, C. gregata, M. phaseolina*, *and P. pachyrhizi*, respectively (Fig. 2)*.* Only three genes were shared between species, revealing a high specificity in plant-pathogen interactions for these species. The three genes are shared by *F. virguliforme* and *F. graminearum*, suggesting that some conservation can occur at the genus level, but not at other broader taxonomic levels.

The specificity of resistance genes to particular species has been widely reported^26–29^. This phenomenon imposes a challenge for biotechnological applications, as it requires pyramiding many different genes to render elite cultivars resistant to different pathogens. However, we cannot rule out that the species-specific trend we observed results from low diversity in the association panels in the GWAS we analyzed. Additionally, as SNP and transcriptome data are not available for multiple pathogen strains, we might overlook broad-spectrum resistance genes that confer resistance to multiple strains of the same species^27^.

**Figure 3:**
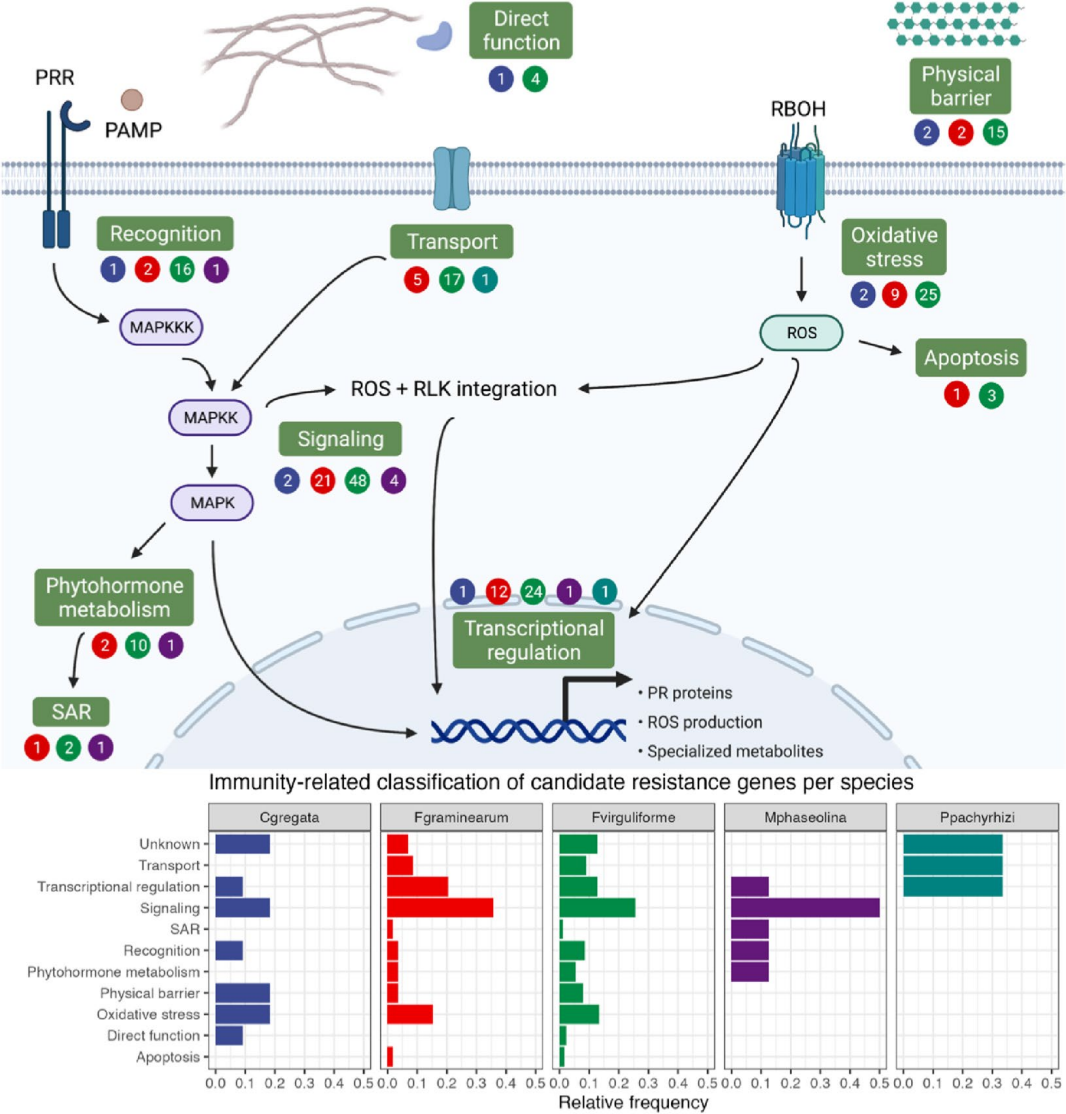
Prioritized candidate resistance genes and their putative role in plant immunity. Numbers in circles represent absolute frequencies of resistance genes against *C. gregata* (blue)*, F. graminearum* (red)*, F. virguliforme* (green)*, M. phaseolina* (purple)*,* and *P pachyrhizi* (turquoise). PRR, pattern recognition receptor. PAMP, pathogen-associated molecular pattern. MAPKKK, mitogen-activated protein kinase kinase kinase. MAPKK, mitogen-activated protein kinase kinase. MAPK, mitogen-activated protein kinase. SAR, systemic acquired resistance. RBOH, respiratory burst oxidase homolog. ROS, reactive oxygen species. RLK, receptor-like kinase. PR, pathogenesis-related. Figure designed with Biorender (biorender.com).

Further, we manually curated the high-confidence candidate resistance genes to predict the putative role of their products in plant immunity (Supplementary Table S4). Most of the prioritized candidates (28%) encode proteins involved in immune signaling, although this does not apply to all fungi species (Fig. 3). The main discrepancy in the functional classification of candidates was observed for candidate resistance genes against *P. pachyrhizi.* However, this is likely due to sampling bias, as the number of SNPs associated with resistance to *P. pachyrhizi* is limited as compared to other species. Candidates also encode proteins that play a role in recognition, phytohormone metabolism, systemic acquired resistance, transport, transcriptional regulation, oxidative stress, apoptosis, physical defense, and direct function against fungi (Fig. 3).

Interestingly, 21 candidate genes lack functional description and, hence, we could not infer their roles in plant immunity (*n* = 2, 4, 14, and 1 for *C. gregata, F. virguliforme,* and *P. pachyrhizi*, respectively). Nevertheless, as they were identified as high-confidence candidate genes, we hypothesize that they encode defense-related proteins. This finding reveals that besides the identification of high-confidence candidate genes, our algorithm can serve as a network-based approach to predict functions of unannotated genes, similar to previous approaches^30,31^.

We also developed a scheme that was used to rank high-confidence candidate genes (Table 2). Ranking candidates is particularly useful to prioritize genes when there are several candidates, such as for *F. virguliforme* and *F. graminearum*. Here, we suggest using the top 10 candidate resistance genes against each pathogen for experimental validation in future studies. Experimental tests with transgenic or edited soybeans using our set of target genes will likely reveal which genes are more suitable to develop soybean lines with increased resistance to each fungal disease.

**Table 2:** Top 10 candidate resistance genes against each fungal species and their putative roles in plant immunity.

Gene	Predicted function	Resistance to	Role
Glyma.16G170100	Cell wall biogenesis-related extensin 3	*C. gregata*	Physical barrier
Glyma.02G026700	Transcriptional repressor SIN3	*C. gregata*	Transcriptional regulation
Glyma.02G026900	Galacturonosyltransferase	*C. gregata*	Physical barrier
Glyma.02G029300	SAM domain-containing	*C. gregata*	Unknown
Glyma.16G155100	Aquaporin	*C. gregata*	Oxidative stress
Glyma.17G217000	Class V chitinase	*C. gregata*	Direct function
Glyma.17G213600	Calcium-binding EF hand	*C. gregata*	Signaling
Glyma.17G231800	Clathrin adaptor EPSIN1	*C. gregata*	Recognition
Glyma.02G047000	Thiosulfate sulfurtransferase/rhodanese	*C. gregata*	Oxidative stress
Glyma.16G150500	Unknown	*C. gregata*	Unknown
Glyma.17G087500	SOUL heme-binding protein	*F. graminearum*	Oxidative stress
Glyma.06G121300	GRAS transcription factor	*F. graminearum*	Transcriptional regulation
Glyma.05G070300	Tobamovirus multiplication 2A	*F. graminearum*	Recognition
Glyma.04G013500	BURP domain-containing protein	*F. graminearum*	Physical barrier
Glyma.06G105000	ERF/AP2 transcription factor	*F. graminearum*	Transcriptional regulation
Glyma.05G062400	2OG-Fe(II) oxygenase	*F. graminearum*	Oxidative stress
Glyma.05G063600	ERF/AP2 transcription factor	*F. graminearum*	Transcriptional regulation
Glyma.05G115700	RING domain ubiquitin E3 ligase	*F. graminearum*	Signaling
Glyma.17G116100	MAPK signaling-related protein	*F. graminearum*	Signaling
Glyma.05G103600	Peroxidase	*F. graminearum*	Oxidative stress
Glyma.13G081000	Nodulin-like amino acid transporter	*F. virguliforme*	Transport
Glyma.01G225600	Unknown	*F. virguliforme*	Unknown
Glyma.02G210500	bHLH transcription factor	*F. virguliforme*	Transcriptional regulation
Glyma.01G162500	BIG1 protein	*F. virguliforme*	Apoptosis
Glyma.17G061400	Peroxidase	*F. virguliforme*	Oxidative stress
Glyma.19G010100	HD-Zip transcription factor	*F. virguliforme*	Transcriptional regulation
Glyma.18G276800	Amino acid transporter	*F. virguliforme*	Oxidative stress
Glyma.05G209900	PLAC8 family protein	*F. virguliforme*	Apoptosis
Glyma.14G025100	Inositol-1,4,5-trisphosphate 5-phosphatase	*F. virguliforme*	Signaling
Glyma.19G117800	Unknown	*F. virguliforme*	Unknown
Glyma.20G203900	Type I serine/threonine protein phosphatase	*M. phaseolina*	Signaling
Glyma.08G316500	Calmodulin-dependent protein kinase	*M. phaseolina*	Signaling
Glyma.06G187200	R-gene-mediated resistance, lipase	*M. phaseolina*	SAR
Glyma.09G218600	Cytochrome P450, family 707, subfamily A	*M. phaseolina*	Phytohormone metabolism
Glyma.09G216800	Pectin acetylesterase	*M. phaseolina*	Signaling
Glyma.20G216600	Dof-type transcription factor	*M. phaseolina*	Transcriptional regulation
Glyma.08G332800	Calcineurin B-like calcium sensor	*M. phaseolina*	Signaling
Glyma.18G301700	Leucine-rich repeat receptor kinase (LRR-RK)	*M. phaseolina*	Recognition
Glyma.15G125900	Magnesium transporter CorA-like	*P. pachyrhizi*	Transport
Glyma.18G286900	Unknown	*P. pachyrhizi*	Unknown
Glyma.15G123900	CBF1 interacting co-repressor CIR	*P. pachyrhizi*	Transcriptional regulation

The predicted function for each gene was manually curated from the description of the best ortholog in *Arabidopsis thaliana*, using functional annotations from Soybase and TAIR.

### Pangenome presence/absence variation analysis demonstrates that most prioritized genes are core genes

We analyzed PAV patterns for our prioritized candidate genes in the recently published pangenome of cultivated soybeans to unveil which soybean genotypes contain prioritized candidate genes and explore gene presence/absence variation patterns across genomes^18^. We found that most candidates are present in all 204 accessions (Supplementary Fig. 1A). This trend is not surprising, as the gene content in this pangenome is highly conserved, with ~ 91% of the genes being shared by > 99% of the genomes. Although the variable genome is enriched in genes associated with defense, signaling, and plant development, this trend was not found in our gene set.

Further, we investigated if gene PAV patterns could be explained by the geographical origins of the accessions (Supplementary Fig. 1B). We observed no clustering by geographical origin, suggesting that gene PAV is not affected by population structure. As this pangenome is comprised of improved soybean accessions^18^, the lack of population structure effect can be due to breeding programs targeting optimal adaptation to different environmental conditions (e.g., latitude and climate), even if they are in the same country.

### Screening of the USDA germplasm reveals a room for genetic improvement

We inspected the USDA germplasm to find the top 5 most resistant genotypes against each fungal pathogen (see Materials and Methods for details). Strikingly, the most resistant genotypes do not contain all resistance alleles, revealing that, theoretically, they could be further improved to increase resistance (Table 3). All resistance-associated SNPs against *P. pachyrhizi* are present in some accessions, but this is because only two SNPs have been reported for this species. Additionally, none of the reported SNPs for *F. graminearum* have been identified in the SoySNP50k collection. Hence, we could not predict the most resistant accessions to this fungal species in the USDA germplasm.

**Table 3:** Top 5 most resistant soybean accessions against each fungal pathogen.

Accession	Score	Potential	Species
PI594466	102	0.73	*C. gregata*
PI578477A	100	0.71	*C. gregata*
PI437571	100	0.71	*C. gregata*
PI567520A	100	0.71	*C. gregata*
PI274507	100	0.71	*C. gregata*
PI339871C	82	0.60	*F. virguliforme*
PI378694	80	0.59	*F. virguliforme*
PI407145	80	0.59	*F. virguliforme*
PI424107A	80	0.59	*F. virguliforme*
PI479753A	80	0.59	*F. virguliforme*
PI594760B	24	0.75	*M. phaseolina*
PI479752	24	0.75	*M. phaseolina*
PI603706A	24	0.75	*M. phaseolina*
PI603531A	24	0.75	*M. phaseolina*
PI603412A	24	0.75	*M. phaseolina*
PI603547	4	1	*P. pachyrhizi*
PI639559A	4	1	*P. pachyrhizi*
PI639559B	4	1	*P. pachyrhizi*
PI326582A	4	1	*P. pachyrhizi*
PI407057	4	1	*P. pachyrhizi*

Overall, the best genotypes do not reach the maximum potential. An exception is observed for *P. pachyrhizi*-resistant genotypes, but this is likely due to the small number of resistance SNPs. None of the resistance SNPs for *F. graminearum* have been identified in the USDA SoySNP50k compendium and, hence, we could not predict resistance potential against this species.

Although some individual genes can confer full race-specific resistance to some pathogens, their durability in the field is often short because of pathogen evolution^27^. Thus, pyramiding quantitative trait loci (QTL) that confer partial resistance has been proposed as a strategy to confer long-term resistance^28^. To accomplish this, the most resistant genotypes identified here can be targets of allele pyramiding in breeding programs using marker-assisted selection. Alternatively, these genotypes might have their genomes edited with CRISPR/Cas systems to introduce beneficial alleles or remove deleterious alleles, ultimately boosting resistance.

### Development of a user-friendly web application for network exploration

**Figure 4:**
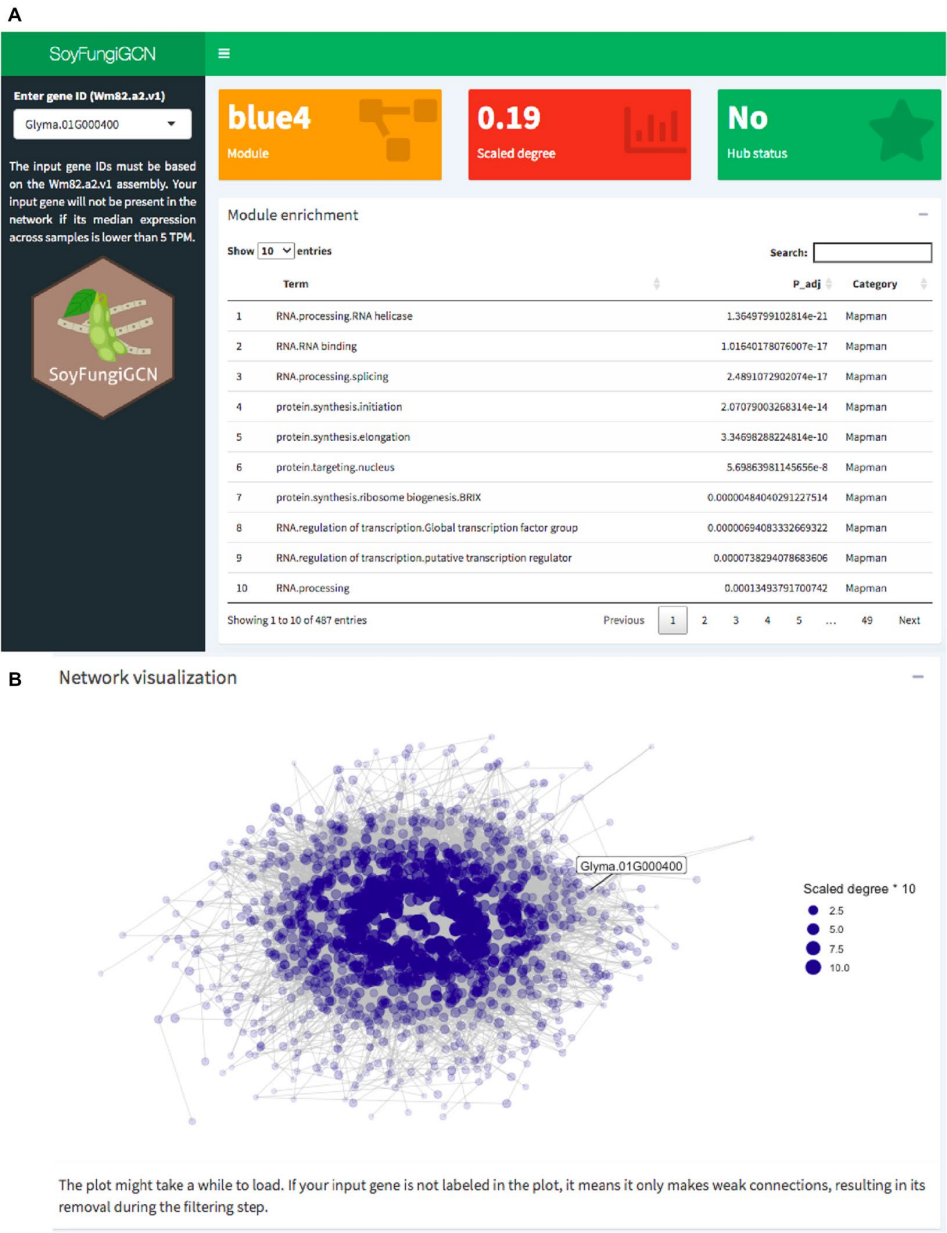
Functionalities in the SoyFungiGCN web application. A. Screenshot of the page users see when they access the application. In the sidebar, users can specify the ID of a gene of interest (Wm82.a2.v1 assembly). For each gene, users can see the gene’s module (orange box), scaled degree (red box), hub gene status (green box), and an interactive table with enrichment results for MapMan bins, Interpro domains and Gene Ontology terms associated the gene’s module. *P* values from enrichment results are adjusted for multiple testing with Benjamini–Hochberg correction. B. Network visualization plot. Users can optionally visualize the input gene and its position in the module by clicking the plus (+) icon in the “Network visualization” tab below the enrichment table. As the plot can take a few seconds to render (~ 2–5 s), it is hidden by default.

To facilitate network exploration and data reuse, we developed a user-friendly web application named SoyFungiGCN (https://soyfungigcn.venanciogroup.uenf.br/). Users can input a soybean gene of interest (Wm82.a2.v1 assembly) and visualize the gene’s module, scaled intramodular degree, and hub status (Fig. 4A). Additionally, users can explore enriched GO terms, Mapman bins and/or Interpro domains associated with the input gene’s module (Fig. 4A). Users can also visualize a network plot with the input gene and its coexpression neighbors (Fig. 4B). This resource can be particularly useful for researchers studying soybean response to other fungal species, as they can check if their genes of interest are located in defense-related coexpression modules. Also, researchers studying other species can verify if the soybean ortholog of their genes of interest is located in a defense-related module. The application is also available as an R package named SoyFungiGCN (https://github.com/almeidasilvaf/SoyFungiGCN). This package lets users run the application locally as a Shiny app, ensuring the application will always be available, even in case of server downtime.

## Conclusions

By integrating publicly available GWAS and RNA-seq data, we found promising candidate genes in soybean associated with resistance to five common phytopathogenic fungi, namely *C. gregata*, *F. graminearum*, *F. virguliforme*, *M. phaseolina,* and *P. pachyrhizi.* The prioritized candidates encode proteins that play a role immunity-related processes such as in recognition, signaling, transcriptional regulation, oxidative stress, and physical defense. We have also found the top 5 most resistant soybean accessions against each fungal species and hypothesize that they can be further genetically improved in breeding programs with marker-assisted selection or through genome editing. The coexpression network generated here was also made available in a web resource and R package to help in future studies on soybean-pathogenic fungi interactions.

## Supplementary Information

Supplementary Figures.

Supplementary Tables.

## Data Availability

All data and code used in this study are available in our GitHub repository (https://github.com/almeidasilvaf/SoyFungi_GWAS_GCN) to ensure full reproducibility.
